# 

**Published:** 1853-06

**Authors:** 


### CONTENTS.

ORIGINAL COMMUNICATIONS:— PAGE

ART. I.—Case of Biliary Calculi, complicated with disease of the Heart. By Ira Manly, Jr. . . . . . . . . . . . . 81
ART. II.—Reports of cases. By Thos. Hall, M.D. . . . . . . 84
ART. III.—Stomatitis Materni. By Alfred S Castleman, M.D. . 88
ART. IV.—A simple arrangement of a Fracture Box for the Leg. By Philip Kirwan, M.D. . . . . . . . . . . . . 90
ART. V.—The Employment of Digitaline in Spormatorrhœa. By M. Lucien Corvisart. . . . . . . . . . . . . . 91
ART. VI.—On the use of Ergot in certain cases of retention of Urine. By M. Passot, of Lyons, . . . . . . . . . 94
An Inquiry, Critical and Experimental, into the Pathology of Fever. By N. S. Davis, M.D. . . . . . . . . . . . . 97

SELECTIONS:—

Clinical Examination of the Urine. By John Locke, M.D. . . 113
A Case of Osteo Aneurism (?) occurring in the Os Calcis; amputation and Recovery. By W. Parker, M.D. . . . . . . . . 118
Color Blindness. . . . . . . . . . . . . 120
Duty of Medical men. . . . . . . . . . . . 121
Nitrate of Silver in Dyspepsia or Chronic Gastritis. . . . . . 122
On Hemorrhage. On a New Styptic. By M. Pagliari. . . . 122
On Reflex Phenomena. By M. C. Bernard. . . . . . . 123
Medical Teaching in Paris, &c. By D. J. Cain, M.D. . . . . 125

EDITORIAL:—

Minutes of the Third Annual Meeting of the Illinois State Medical Society, held at Chicago, June, 1853. . . . . . . . . . . 131
North-Western Medical Society. . . . . . . . . . 142

### RECEIPTS
### FOR THE JOURNAL DURING THE MONTH OF MAY.

**ILLINOIS.**

